# Circulating Vascular Adhesion Protein-1 Level Predicts the Risk of Cardiovascular Events and Mortality in Hemodialysis Patients

**DOI:** 10.3389/fcvm.2021.701079

**Published:** 2021-09-07

**Authors:** Dae Kyu Kim, Yu Ho Lee, Jin Sug Kim, Yang Gyun Kim, So-Young Lee, Shin Young Ahn, Dong-Young Lee, Kyung Hwan Jeong, Sang-Ho Lee, Hyeon Seok Hwang, Ju-Young Moon

**Affiliations:** ^1^Department of Medicine, Graduate School, Kyung Hee University, Seoul, South Korea; ^2^Division of Nephrology, Department of Internal Medicine, CHA Bundang Medical Center, CHA University, Seongnam, South Korea; ^3^Division of Nephrology, Department of Internal Medicine, Kyung Hee University, Seoul, South Korea; ^4^Division of Nephrology, Department of Internal Medicine, Korea University College of Medicine, Seoul, South Korea; ^5^Division of Nephrology, Department of Internal Medicine, Veterans Health Service Medical Center, Seoul, South Korea

**Keywords:** vascular adhesion protein-1, hemodialysis, cardiovascular disease, endothelial dysfunction, diastolic dysfunction

## Abstract

**Background:** Vascular adhesion protein-1 (VAP-1) is an oxidative enzyme of primary amines that facilitates the transmigration of inflammatory cells. Its oxidative and inflammatory effects are prominently increased in pathological conditions, such as metabolic, atherosclerotic, and cardiac diseases. However, the clinical significance of circulating VAP-1 levels in hemodialysis (HD) patients is unclear.

**Methods:** A total of 434 HD patients were enrolled in a prospective multicenter cohort study between June 2016 and April 2019. Plasma VAP-1 levels were measured at the time of data entry, and the primary endpoint was defined as a composite of cardiovascular (CV) and cardiac events.

**Results:** Circulating VAP-1 levels were positively correlated with plasma levels of cardiac remodeling markers, including brain natriuretic peptide, galectin-3, and matrix metalloproteinase-2. Multivariable logistic regression analysis revealed that patients with higher circulating VAP-1 levels were more likely to have left ventricular diastolic dysfunction [odds ratio, 1.40; 95% confidence interval [CI], 1.04–1.88]. The cumulative event rate of the composite of CV events was significantly greater in VAP-1 tertile 3 than in VAP-1 tertiles 1 and 2 (*P* = 0.009). Patients in tertile 3 were also associated with an increased cumulative event rate of cardiac events (*P* = 0.015), with a 2.06-fold higher risk each for CV (95% CI, 1.10–3.85) and cardiac (95% CI, 1.03–4.12) events after adjusting for multiple variables.

**Conclusions:** Plasma VAP-1 levels were positively associated with left ventricular diastolic dysfunction and the risk of incident CV and cardiac events in HD patients. Our results indicate that VAP-1 may aid clinicians in identifying HD patients at a high risk of CV events.

## Introduction

Patients receiving hemodialysis (HD) have substantial retention of uremic toxins, which lead to a number of adverse metabolic processes (1). Oxidative stress and inflammation are representative pathophysiologic processes of uremic complications and are major contributors of cardiovascular (CV) complications in HD patient (2–5), by promoting myocardial stiffening and left ventricular (LV) hypertrophy and inducing endothelial dysfunction and progression of atherosclerosis (5–7). These conditions severely increase the risk of CV complications, which have become leading causes of death in HD patients (8).

Vascular adhesion protein-1 (VAP-1) is a semicarbazide-sensitive amine oxidase that catalyzes the oxidative deamination of primary amines, which generates free radicals and causes oxidative stress (9, 10). It also facilitates the transmigration of inflammatory cells and worsens injuries in inflamed tissues (9, 11). These deleterious roles are prominently enhanced in pathological conditions. Circulating VAP-1 levels are increased in septic, metabolic, and autoimmune diseases, and higher VAP-1 levels increase the risk of atherosclerotic events and CV mortality (12–14). In patients with impaired renal function, excessive concentrations and abundant substrates of VAP-1 were observed, the latter of which undergo uncontrolled deamination and oxidative stress (14, 15). Furthermore, VAP-1 is suggested to play a pivotal role in HD patients because many dialysis-specific factors upregulate inflammatory processes (15, 16). Therefore, VAP-1 may be critically involved in the occurrence of adverse CV events in HD patients through oxidative stress and inflammation.

However, the clinical significance of VAP-1 has rarely been evaluated in HD patients, and no reports have investigated the prognostic significance of VAP-1. In this study, we investigated the association between circulating VAP-1 levels and risk of incident adverse CV events in HD patients, along with the relationship of echocardiographic parameters and circulating cardiac biomarkers with VAP-1 levels.

## Materials and Methods

### Study Population

All data in this study were obtained from the K-cohort registry, which is a multicenter, internet-based, prospective cohort of HD patients in Korea designed to investigate the prognostic markers of CV complications and mortality. Patients from six general hospitals were enrolled if they were aged >18 years and received regular 4-h HD prescriptions per session that occurred thrice a week for at least 3 months. The exclusion criteria were as follows: pregnancy, hematological malignancy, presence of a solid tumor, and a life expectancy of <6 months. A total of 637 patients were recruited between June 2016 and April 2019, and 434 patients with whole plasma samples at the time of enrollment were included. The study protocol was approved by the local ethics committee (KHNMC 2016-04-039), and the study was conducted in accordance with the principles of the Declaration of Helsinki. All involved participants signed written informed consent forms before enrollment.

The patients were classified into three groups based on the circulating VAP-1 levels as follows: tertile 1, <343.2 ng/mL; tertile 2, 343.2– <438.2 ng/mL; and tertile 3, ≥438.2 ng/mL. All patients were prospectively followed up for specific clinical events after baseline assessments. Patient follow-up was censored at the time of transfer to peritoneal dialysis, kidney transplantation, loss of follow-up, or patient consent withdrawal.

### Data Collection and Outcome Measures

Information on baseline demographic factors, laboratory data, dialysis, and concomitant medications were collected from medical records and interviews. Information on comorbidities were investigated and used to calculate the Charlson comorbidity index score (17). Fasting blood samples for laboratory data and enzymatic measurements were collected before the start of HD in a midweek session.

The primary endpoint was a composite of incident CV events and mortality, which included cardiac events such as coronary artery disease requiring coronary artery bypass surgery or percutaneous intervention, myocardial infarction, heart failure, ventricular arrhythmia, cardiac arrest, and sudden death, as well as cerebral infarction, cerebral hemorrhage, and peripheral vascular occlusive diseases requiring revascularization or surgical intervention. All-cause mortality events were recorded. The secondary endpoints were the correlation of VAP-1 levels with LV diastolic dysfunction, which was defined as peak early diastolic flow velocity (E)/peak early diastolic tissue velocity (E') of >15 on echocardiography, and levels of circulating cardiac markers.

### Echocardiographic Measurements

Among the enrolled patients, 214 (49.3%) underwent echocardiography [61 (42.4%) in tertile 1, 75 (51.7%) in tertile 2, and 78 (53.8%) in tertile 3]. Cardiologists and trained sonographers examined two-dimensional and M-mode echocardiographs based on the recommendations of the American Society of Echocardiography (18). LV end-diastolic diameter (LVDd), LV end-systolic diameter, LV posterior wall thickness, and interventricular septal thickness were measured in the M-mode echocardiogram. LV mass was estimated using the Devereux formula, with the body surface area as the index. LV end-diastolic and LV end-systolic volumes, LV ejection fraction, and left atrial dimensions were determined in apical two- and four-chamber views. E and peak late diastolic flow velocity (A) was determined from the mitral valve inflow velocity curve using pulsed-wave Doppler ultrasonography. E′ was measured from the septal aspect of the mitral annulus using tissue Doppler. The E/A and E/E′ ratios were calculated.

### Measurements of Circulating Cardiac Markers and VAP-1 Levels

Baseline plasma samples for the measurement of N-terminal pro-B-type natriuretic peptide (NT-proBNP), brain natriuretic peptide (BNP), matrix metalloproteinase-2 (MMP-2), and VAP-1 were collected using ethylenediaminetetraacetic acid-treated tubes. After centrifugation for 15 min at 1,000 × g at room temperature, the samples were stored at 80°C until use. Enzyme-linked immunosorbent assay was performed using Magnetic Luminex® Screening Assay multiplex kits (R&D Systems, Inc., Minneapolis, MN, USA).

### Statistical Analysis

Data are expressed as means ± standard deviations (SDs) or medians (interquartile ranges). Differences among the three groups were identified using analysis of variance or Kruskal-Wallis test. Tukey *post-hoc* test and Mann-Whitney *U*-test with Bonferroni correction were used to identify intergroup differences. Chi-square test or Fisher's exact test was used to compare the categorical variables. Log-transformed high-sensitivity C-reactive protein (hsCRP) values were used because of the skewed data distribution. Correlation between the VAP-1 levels and continuous variables was evaluated using Spearman's analyses. Binary logistic regression analysis was used to assess the association between the VAP-1 levels and LV diastolic dysfunction. A Cox proportional hazards model was constructed to identify independent variables related to CV and cardiac events and all-cause mortality. Parameters significantly associated with weight in the univariable analysis and clinically fundamental parameters were included in the multivariable models. Formal tests for the interaction between VAP-1 levels and predefined subgroups were conducted in addition to the main effects of the fully adjusted models. We modeled the association between VAP-1 levels and the hazard ratio to predict CV events. We used three knots and restricted cubic spline transformations to continuous measures. We calculated the sample size using standard formulas based on the number of patients to obtain the adequate statistical power for the primary endpoint and show a different composite event-free survival rate with an α-level of 0.05, β error of 0.20, and hazard ratio of 1.5. The minimum required sample size in each group was 98 patients. Statistical significance was set at *P* < 0.05. Statistical analyses were performed using the SPSS software (version 22.0; SPSS, IBM Corp., Armonk, NY, USA) and R software (version 3.6.2).

## Results

### Baseline Demographic Characteristics and Laboratory Data

The mean VAP-1 level was 386.0 (range, 318.1–484.6) ng/mL, with mean VAP-1 levels in tertiles 1, 2, and 3 of 281.8 ng/mL (range, 242.6–318.5 ng/mL), 385.5 ng/mL (range, 365.6– 411.0 ng/mL), and 523.6 ng/mL (range, 484.6–601.4 ng/mL), respectively. The baseline clinical characteristics, demographics, and laboratory results are shown in Table 1. Patients in tertile 3 had a shorter HD history, higher prevalence of diabetes mellitus, higher Charlson comorbidity index, higher pre-dialysis systolic blood pressure (SBP), and lower intact parathyroid hormone (i-PTH) than those in tertile 1. Regarding the circulating cardiac markers, patients in tertile 3 showed the highest BNP and MMP-2 levels. Plasma MMP-2 levels were moderately correlated with plasma VAP-1 levels, whereas BNP and galectin-3 showed weak positive correlations (Supplementary Table S1). In contrast, hsCRP levels demonstrated a weak negative correlation with circulating VAP-1 levels.

**Table 1:** Baseline demographic and laboratory data of the study population.

	**Tertiles of circulating VAP-1 levels**			
	**Tertile 1 (<343.2 ng/mL)**	**Tertile 2 (343.2–438.2 ng/mL)**	**Tertile 3 (≥438.2 ng/mL)**	***P*-value**
Age (years)	60.5 ± 14.9	63.0 ± 12.1	61.6 ± 11.2	0.262
Males, n (%)	93 (64.6)	102 (70.3)	96 (66.2)	0.561
Body mass index (kg/m^2^)	23.3 ± 4.1	23.1 ± 3.7	23.5 ± 4.5	0.725
HD duration (years)	4.4 ± 5.8	4.1 ± 5.6	2.7 ± 4.1^a, b^	0.014
History of CV events, n (%)	81 (84)	20 (86)	18 (87)	0.747
Diabetes mellitus, n (%)	45 (31.7)	82 (56.6)^a^	120 (82.8)^a, b^	<0.001
Charlson comorbidity index	3.6 ± 1.6	4.2 ± 1.6^a^	4.5 ± 1.2^a^	<0.001
Pre-dialysis SBP (mm Hg)	138.7 ± 20.8	142.0 ± 19.0	147.0 ± 20.1^a^	0.002
Hemoglobin (g/dL)	10.4 ± 1.4	10.6 ± 1.1	10.3 ± 1.1	0.338
Glucose (mg/dL)	139.3 ± 57.9	150.1 ± 61.9	172.6 ± 66.5^a, b^	<0.001
Albumin (g/dL)	3.8 ± 0.4	3.8 ± 0.3	3.8 ± 0.3	0.515
LDL-C (mg/dL)	79.0 ± 26.9	75.4 ± 26.5	74.3 ± 23.7	0.276
hsCRP (mg/dL)	1.4 (0.2–5.5)	0.8 (0.2–2.9)	0.9 (0.2–2.7)	0.062
i-PTH (pg/mL)	287.0 ± 244.1	292.5 ± 232.3	233.9 ± 165.0^a, b^	0.040
spKt/V	1.6 ± 0.5	1.6 ± 0.3	1.5 ± 0.3	0.219
Catheter as vascular access, *n* (%)	8 (5.6)	5 (3.4)	7 (4.8)	0.679
Follow-up years	30.8 ± 14.8	29.9 ± 14.4	29.9 ± 14.0	0.823
NT-proBNP (pg/mL)	286 (165–466)	311 (207–431)	335 (226–508)	0.076
BNP (pg/mL)	33.4 (7.6–68.4)	33.4 (6.7–88.3)	55.6 (13.4–108.2)^a^	0.015
Galectin-3 (ng/mL)	16.8 (15.0–20.0)	17.8 (15.1–20.6)	18.2 (15.3–21.4)	0.057
MMP-2 (ng/mL)	577 (478–678)	665 (568–763)	746 (626–897)^a, b^	<0.001

*Data are expressed as means ± standard deviations or medians (interquartile ranges), unless otherwise specified. VAP, vascular adhesion protein; HD, hemodialysis; CV, cardiovascular; LDL-C, low-density lipoprotein cholesterol; i-PTH, intact parathyroid hormone; hsCRP, high-sensitivity C-reactive protein; SBP, systolic blood pressure; NT-proBNP, N-terminal pro-B-type natriuretic peptide; BNP, brain natriuretic peptide; MMP, matrix metalloproteinase.*

*^a^P < 0.05 vs. tertile 1.*

*^b^P < 0.05 vs. tertile 2*.

### Relationship Between Plasma VAP-1 Levels and LV Diastolic Dysfunction in Hemodialysis Patients

Baseline echocardiographic measurements are presented in Supplementary Table S2. LVDd was significantly different among the tertiles, with the highest E wave and E/A and E/E' ratios observed in patients in VAP-1 tertile 3. We constructed univariable and multivariable binary logistic regression models to determine the association between VAP-1 and LV diastolic dysfunction (Table 2). In the univariable analysis, circulating VAP-1 level increment per SD [dds ratio [OR], 1.51; 95% confidence interval [CI], 1.15–2.00; *P* = 0.004] and Charlson comorbidity index (OR, 1.32; 95% CI, 1.08–1.62; *P* = 0.006) were significantly associated with an increased risk of LV diastolic dysfunction. Age, male sex, pre-dialysis SBP, and NT-proBNP increments per SD showed a borderline significant association with LV diastolic dysfunction. In the multivariable binary logistic regression model, the Charlson comorbidity index (OR, 1.24; 95% CI, 1.01–1.53; *P* = 0.045) and serum VAP-1 level (OR, 1.40; 95% CI, 1.04–1.88; *P* = 0.028) remained statistically significant as independent determinants of LV diastolic dysfunction in HD patients.

**Table 2:** Relationship between vascular adhesion protein-1 levels and left ventricle diastolic dysfunction.

	**Univariable analysis**		**Multivariable analysis**	
	**OR (95% CI)**	***P*-value**	**OR (95% CI)**	***P*-value**
Age	1.02 (1.00–1.04)	0.057	1.02 (1.00–1.05)	0.078
Male sex	0.59 (0.33–1.04)	0.066	0.58 (0.32–1.07)	0.083
BMI	1.01 (0.94–1.07)	0.880	–	–
Hemodialysis duration	1.04 (0.96–1.19)	0.362	–	–
Charlson comorbidity index	1.32 (1.08–1.62)	0.006	1.24 (1.01–1.53)	0.045
Pre-dialysis SBP	1.01 (1.00–1.03)	0.063	1.01 (0.99–1.02)	0.327
Hemoglobin	1.08 (0.85–1.37)	0.544	–	–
Albumin	0.62 (0.26–1.48)	0.281	–	–
LDL-C	1.00 (0.99–1.01)	0.365	–	–
hsCRP	1.05 (0.75–1.48)	0.765	–	–
NT-proBNP per SD	1.28 (0.95–1.73)	0.109	1.15 (0.83–1.60)	0.393
VAP-1 per SD	1.51 (1.15–2.00)	0.004	1.40 (1.04–1.88)	0.028

*OR, odds ratio; CI, confidence interval; BMI, body mass index; LDL-C, low-density lipoprotein cholesterol; hsCRP, high-sensitivity C-reactive protein; NT-proBNP, N-terminal pro-B-type natriuretic peptide; SD, standard deviation; VAP, vascular adhesion protein*.

### Prognostic Utility of the VAP-1 Level in Hemodialysis Patients

**Figure 1:**
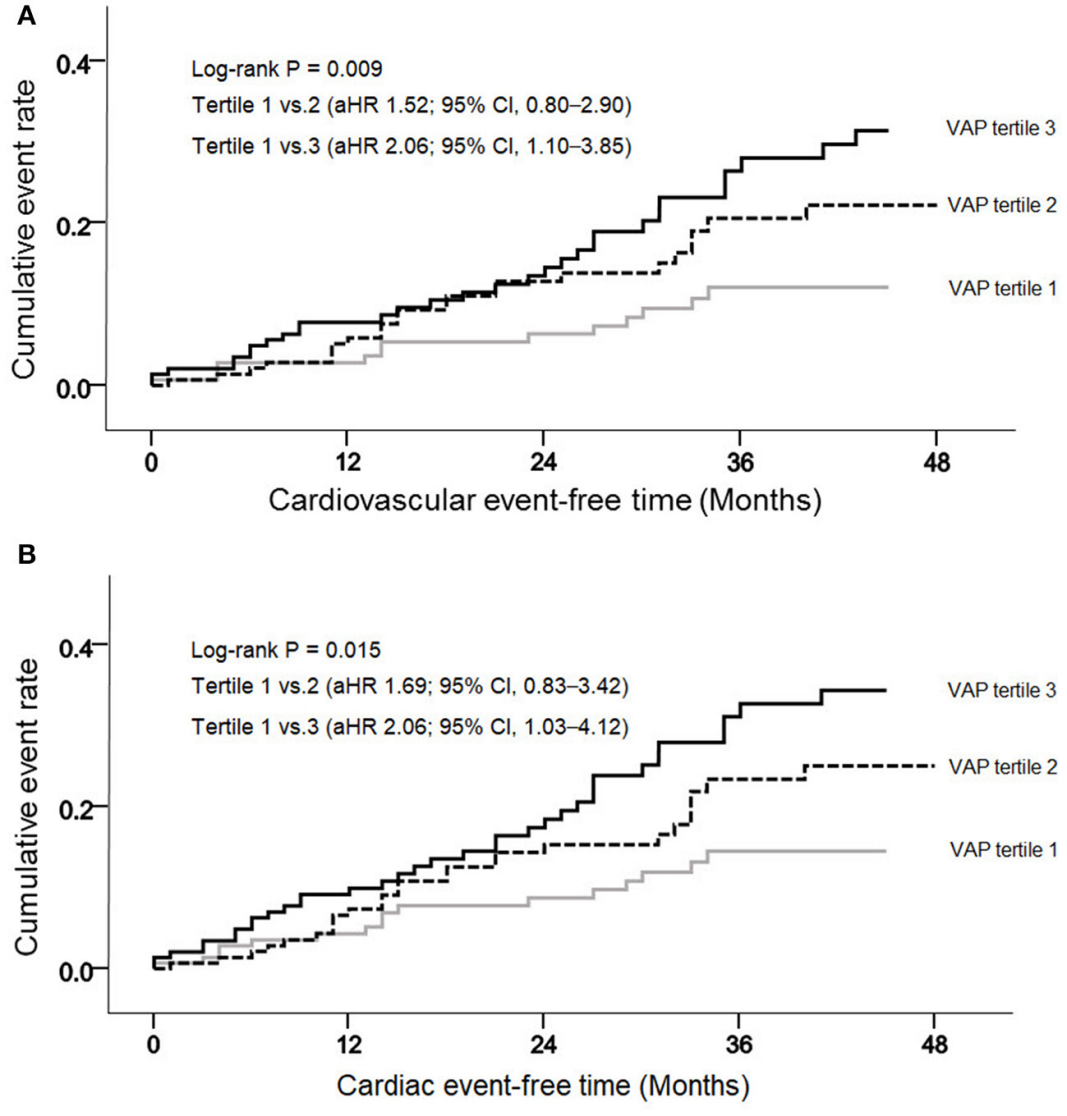
Cumulative cardiovascular **(A)** and cardiac **(B)** event rates, according to the vascular adhesion protein-1 (VAP-1) levels.

During a mean follow-up of 30.3 months, 61 deaths (14.1%) and 77 adverse CV events (17.7%) occurred. Regarding CV events, coronary artery disease occurred in 36 patients, heart failure in 7, ventricular arrhythmia in 1, cardiac arrest in 9, sudden death in 9, CV events in 9, and peripheral vascular occlusive diseases in 6. VAP-1 tertile 3 had the highest cumulative CV event rate (*P* = 0.009; Figure 1A) and a greater cumulative rate of cardiac events (*P* = 0.015; Figure 1B). The cumulative event rate of patient mortality did not differ among VAP-1 tertiles (*P* = 0.747).

**Figure 2:**
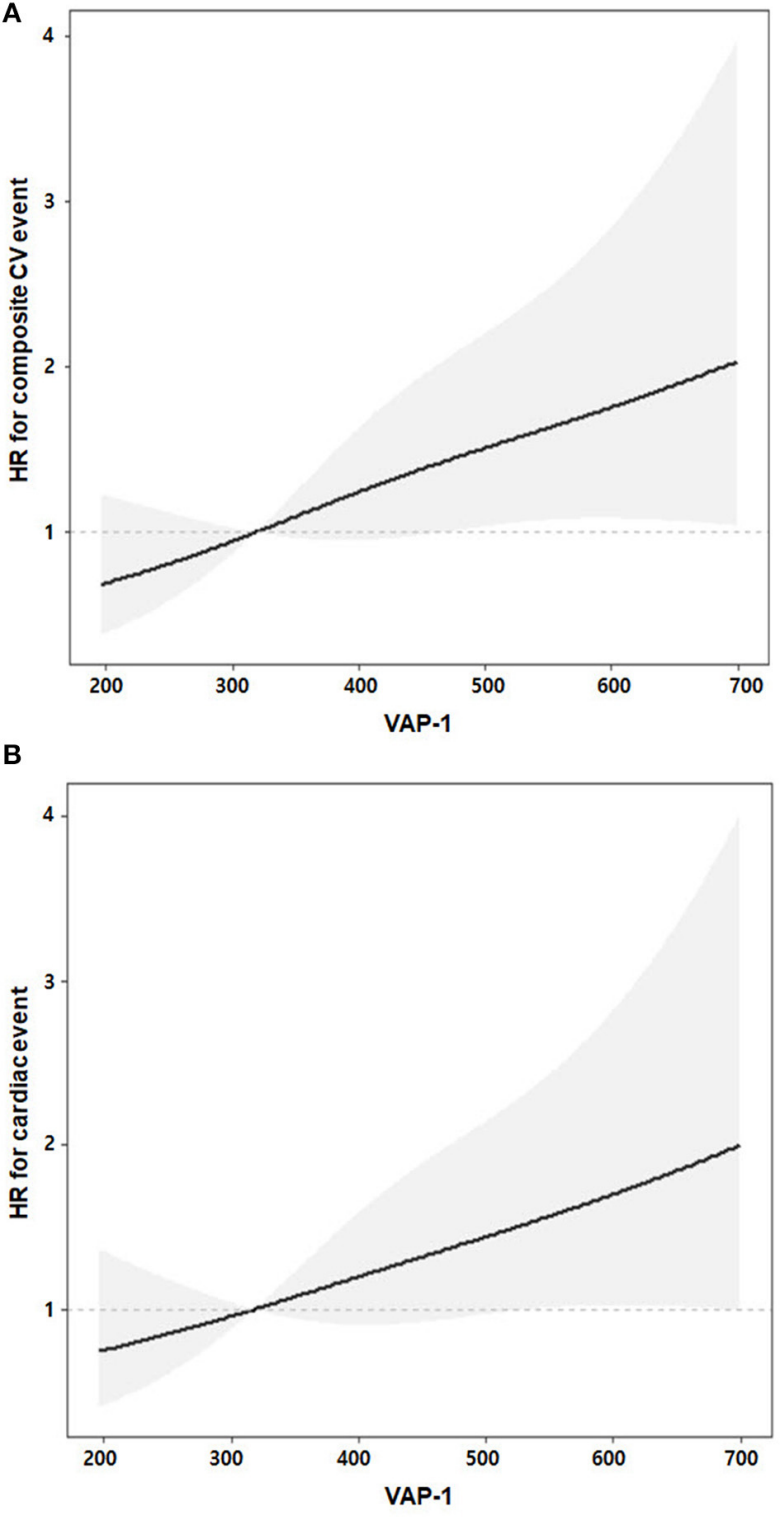
Linear associations of VAP-1 and adjusted risks of the composite of cardiovascular **(A)** and cardiac **(B)** events. Gray shaded areas represent 95% confidence intervals. The adjusted multiple variables were age, sex, body mass index, hemodialysis duration, Charlson comorbidity index, pre-dialysis systolic blood pressure, hemoglobin, low-density lipoprotein cholesterol, high-sensitivity C-reactive protein, spKt/V, catheter use, and N-terminal pro-B-type natriuretic peptide.

Univariable Cox regression analysis revealed that VAP-1 tertile 3 was significantly associated with an increased risk of the composite of CV events [hazard ratio [HR], 2.51; 95% CI, 1.39–4.54; *P* = 0.002] (Table 3). In the multivariable Cox regression analysis, VAP-1 tertile 3 was significantly associated with a 2.06-fold higher risk of CV events (95% CI, 1.10–3.85; *P* = 0.025) and VAP-1 increment per SD was significantly associated with a 1.31-fold higher risk of CV events (95% CI, 1.05–1.64; *P* = 0.019). The risk of cardiac events and patient mortality was further investigated. Patients in VAP-1 tertile 3 had a 2.06-fold higher risk of cardiac disease after adjustment for multiple variables (95% CI, 1.03–4.12; *P* = 0.041). VAP-1 increment per SD was also significantly associated with the risk of cardiac events (HR, 1.29; 95% CI, 1.01–1.64; *P* = 0.038). However, among patients in VAP-1 tertile 3, VAP-1 levels were not significantly associated with the risk of mortality. To evaluate potential linear associations, we evaluated the association between VAP-1 and the risk of composite of CV events and cardiac events during follow-up. The restricted cubic spline model after multiple adjustments showed gradually increasing HRs for both CV and cardiac events with increasing VAP-1 levels (Figure 2).

**Table 3:** Hazard ratios of plasma vascular adhesion protein-1 levels for cardiovascular events, cardiac events, and mortality.

	**Number of**	**Univariable**	**Multivariable**
		**analysis**	**analysis**
	**events (%)**	**HR (95% CI)**	**HR (95% CI)**
*Composite of CV events*			
VAP-1 tertile 1	16 (11.1)	Reference	
VAP-1 tertile 2	26 (17.9)	1.74 (0.93–3.24)	1.52 (0.80–2.90)
VAP-1 tertile 3	35 (24.1)	2.51 (1.39–4.54)	2.06 (1.10–3.85)
VAP-1 per SD	–	1.40 (1.14–1.71)	1.31 (1.05–1.64)
*Cardiac events*			
VAP-1 tertile 1	13 (9.0)	Reference	
VAP-1 tertile 2	23 (15.9)	1.90 (0.96–3.75)	1.69 (0.83–3.42)
VAP-1 tertile 3	30 (20.7)	2.66 (1.38–5.10)	2.06 (1.03–4.12)
VAP-1 per SD	–	1.42 (1.14–1.77)	1.29 (1.01–1.64)
*Patient deaths*			
VAP-1 tertile 1	23 (16.0)	Reference	
VAP-1 tertile 2	20 (13.8)	0.90 (0.50–1.64)	0.85 (0.44–1.65)
VAP-1 tertile 3	18 (12.4)	0.81 (0.44–1.50)	0.86 (0.43–1.72)
VAP-1 per SD	–	0.96 (0.74–1.26)	0.98 (0.72–1.33)

*HR, hazard ratio; CI, confidence interval; CV, cardiovascular; SD, standard deviation; VAP, vascular adhesion protein. All analyses were adjusted for the following covariates: age, sex, body mass index, hemodialysis duration, Charlson comorbidity index, pre-dialysis systolic blood pressure, hemoglobin, low-density lipoprotein cholesterol, high-sensitivity C-reactive protein, spKt/V, catheter use, and N-terminal pro-B-type natriuretic peptide*.

The relationship between VAP-1 levels and the composite of incident CV events was further investigated in subgroups stratified by the presence of diabetes mellitus and NT-proBNP levels, with cut-offs defined as median values in each parameter (>386.5 ng/mL for VAP-1 and >313.3 pg/mL for NT-proBNP, respectively; Table 4). Multivariable analysis showed that higher VAP-1 levels were associated with an increased risk of CV events both in patients with (HR, 2.39; 95% CI, 1.14–5.03; *P* = 0.022) and without (HR, 2.57; 95% CI, 1.05–6.30; *P* = 0.039) diabetes mellitus. There was a significant interaction between higher VAP-1 levels and diabetes mellitus in association with CV events (HR, 0.51; 95% CI, 0.29–0.90; *P* for interaction = 0.02). Compared to those with low NT-proBNP levels, patients with high VAP-1 and NT-proBNP levels showed an increased risk of CV events (HR, 1.99; 95% CI, 1.01–3.89; *P* = 0.046), whereas those with isolated high NT-proBNP levels did not show this association. There was no significant interaction between VAP-1 and NT-proBNP levels (HR, 1.57; 95% CI, 0.59–4.18; *P* for interaction = 0.36).

**Table 4:** Hazard ratios of plasma vascular adhesion protein-1 levels for the composite of cardiovascular events according to predefined subgroups.

	**Number of CV events (%)**	**Univariable analysis HR (95% CI)**	**Multivariable analysis HR (95% CI)**	***P* for interaction**
*Diabetes mellitus*				0.02
Low VAP-1, without DM	11 (8.1)	Reference		
High VAP-1, without DM	9 (17.6)	2.34 (0.97–5.66)	2.57 (1.05–6.30)	
Low VAP-1, with DM	15 (18.5)	2.45 (1.13–5.34)	1.68 (0.73–3.84)	
High VAP-1, with DM	42 (25.3)	3.67 (1.89–7.14)	2.39 (1.14–5.03)	
*NT-proBNP*				0.36
Low VAP-1, low NT-proBNP	14 (12.7)	Reference		
High VAP-1, low NT-proBNP	19 (17.8)	1.66 (0.83–3.30)	1.43 (0.71–2.89)	
Low VAP-1, high NT-proBNP	12 (11.2)	0.97 (0.45–2.09)	0.88 (0.40–1.96)	
High VAP-1, high NT-proNBP	32 (29.1)	2.64 (1.41–4.95)	1.99 (1.01–3.89)	

*CV, cardiovascular; HR, hazard ratio; CI, confidence interval; VAP, vascular adhesion protein; DM, diabetes mellitus; NT-proBNP, N-terminal pro-B-type natriuretic peptide. High VAP-1 was defined as median value > 386.5 ng/mL, and criteria for predefined subgroups were also based on medians; high NT-proBNP > 313.3 pg/mL. All analyses were adjusted for the following covariates (except for the variable used to define the subgroup in each case): age, sex, body mass index, hemodialysis duration, Charlson comorbidity index, pre-dialysis systolic blood pressure, hemoglobin, low-density lipoprotein cholesterol, high-sensitivity C-reactive protein, spKt/V, catheter use, and N-terminal pro-B-type natriuretic peptide*.

## Discussion

In this prospective cohort study, we investigated the associations of plasma VAP-1 levels with cardiac dysfunction and CV outcomes in HD patients. Plasma VAP-1 levels were positively associated with circulating cardiac biomarker levels and LV diastolic dysfunction. Patients in VAP-1 tertile 3 had the greatest risk of a higher composite of CV events, and this association remained significant after adjusting for established CV risk factors. Taken together, our findings suggest that plasma VAP-1 may be a novel biomarker of incident CV events in HD patients.

High blood pressure and hyperglycemia were representative metabolic disorders that increased plasma VAP-1 levels in individuals without renal impairment (13, 19–23). In the present study, serum glucose level and pre-dialysis SBP were the highest among patients in VAP-1 tertile 3, suggesting that the relationship among blood pressure, serum glucose, and VAP-1 levels is consistent in HD patients. Because VAP-1 promoted the inflammatory process, the positive correlation between plasma VAP-1 levels and hsCRP was expected. However, we showed that the correlation between VAP-1 and hsCRP was negative and the correlation power was highly weak, implying that higher plasma VAP-1 levels may not merely be a secondary reflection of systemic inflammation in HD patients.

LV diastolic dysfunction is commonly identified in HD patients and is associated with high CV mortality (24–28). In this study, the E/E' ratio was used as a criterion for LV diastolic dysfunction because an E/E' ratio of >15 has been reported as a strong predictor of CV events in HD patients (29, 30). We found that a higher VAP-1 level was independently associated with an increased risk of LV diastolic dysfunction. In addition, higher VAP-1 levels were correlated with an increased risk of cardiac events after adjusting for multiple confounders, indicating that VAP-1 levels may reflect structural changes in cardiac pathology and that VAP-1 could be a potential biomarker of incident cardiac events in HD patients. Furthermore, we found a significant correlation between plasma VAP-1 and MMP-2, one of the main mediators of pathologic extracellular matrix remodeling and fibrosis in several cardiac diseases (31–33). Previous studies have reported that MMP-2 is involved in LV diastolic dysfunction because circulating MMP-2 levels were correlated with the E/E' ratio (32, 33).

Patients in VAP-1 tertile 3 had a higher cumulative incidence of the composite of CV events than those in VAP-1 tertile 1. In addition, Cox regression analysis showed that high plasma VAP-1 levels were independently associated with a significantly increased risk of CV events, even after adjustments for possible confounders, including SBP and the Charlson comorbidity index. These findings suggest that VAP-1 may be a useful indicator for screening HD patients at a high risk of CV events. Positive correlations of plasma VAP-1 with traditional risk factors, including glucose levels, pre-dialysis SBP, and LV diastolic dysfunction, further support its usefulness in predicting CV outcomes. Notably, plasma VAP-1 levels did not predict all-cause mortality, despite their significant association with CV events. A possible explanation for this discrepancy might be that more than two-third (65.6%) of mortalities in this study were not attributed to CV events.

Subgroup analysis showed that higher plasma VAP-1 levels were associated with an increased risk of CV events in both patients with and without diabetes mellitus. However, the HR of higher VAP-1 levels was lower in HD patients with diabetes mellitus than in HD patients without diabetes mellitus (*P* for interaction = 0.02), indicating that the predictive power of VAP-1 may differ based on the presence of diabetes mellitus. Considering that more HD patients are developing diabetes mellitus (34, 35), a lower predictive power of plasma VAP-1 levels in this subpopulation could be a disadvantage as a prognostic biomarker of CV events. Although the underlying mechanism of this phenomenon could not be assessed in this study, we speculate that hyperglycemia-induced upregulation of unfavorable molecules, such as plasma proinflammatory cytokines and advanced glycation end products, reduce the relative contributions of plasma VAP-1 to the incidence of CV events (36, 37).

The clinical utility of NT-proBNP as a cardiac biomarker in HD patients had been inconclusive, partly because of intra-patient variations and different cut-off values used across studies (38–40). An observational study also reported that elevated NT-proBNP levels are likely caused by intravascular volume expansion rather than cardiac dysfunction in stable HD patients, further complicating the interpretations of its clinical value in HD patients with hypervolemia (41). Our subgroup analysis showed that increased VAP-1 and NT-proBNP levels were associated with a significantly higher risk of composite CV events, whereas isolated NT-proBNP levels were not (Table 4). These findings indicate that plasma VAP-1 levels can help differentiate those at a high risk of adverse CV outcomes among HD patients exhibiting increased baseline NT-proBNP levels.

Our study had some limitations. Analyses for individual CV events could not be performed because of the limited number of events and short follow-up period. Echocardiographic data were obtained in a small proportion of enrolled patients (49.3%), although the analysis of available data revealed an evident association between high plasma VAP-1 levels and LV diastolic dysfunction. Moreover, multivariable analysis might not have controlled all relevant confounding factors, and thus these were not thoroughly assessed in this study.

In conclusion, our study demonstrated that plasma VAP-1 levels were associated with circulating markers of cardiac remodeling, as well as a greater risk of LV diastolic dysfunction. In addition, higher plasma VAP-1 levels were correlated with an increased risk of future CV events in HD patients. Our results indicate that VAP-1 might help overcome the limitations of traditional risk factors in the setting of end-stage renal disease and help clinicians identify HD patients at a high risk of CV events.

## Data Availability Statement

The raw data supporting the conclusions of this article will be made available by the authors, without undue reservation.

## Ethics Statement

The studies involving human participants were reviewed and approved by KyungHee University Hospital IRB. The patients/participants provided their written informed consent to participate in this study.

## Author Contributions

DKK, YHL, and HSH constructed the research questions and designed the analysis. JSK, YGK, S-YL, SYA, D-YL, KHJ, S-HL, and J-YM conducted the data collection. DKK, YHL, J-YM, and HSH drafted the manuscript. All authors reviewed the results, commented on the manuscript, read, and approved the final manuscript.

## Funding

This research was supported by grants from the Patient-Centered Clinical Research Coordinating Center, which was funded by the Ministry of Health & Welfare, Republic of Korea (H19C0481 and HC19C0041).

## Conflict of Interest

The authors declare that the research was conducted in the absence of any commercial or financial relationships that could be construed as a potential conflict of interest.

## Publisher's Note

All claims expressed in this article are solely those of the authors and do not necessarily represent those of their affiliated organizations, or those of the publisher, the editors and the reviewers. Any product that may be evaluated in this article, or claim that may be made by its manufacturer, is not guaranteed or endorsed by the publisher.
