# Supplementary material for: Circulating Vascular Adhesion Protein-1 Level Predicts the Risk of Cardiovascular Events and Mortality in Hemodialysis Patients
Source: Front Cardiovasc Med. 2021 Sep 7;8:701079. doi: 10.3389/fcvm.2021.701079 (PMC8452851; doi:10.3389/fcvm.2021.701079)
Supplement: Supplementary file 1 [file Table_1.DOCX]

Supplementary Material

# Supplementary table

# Table S1 | Correlation between vascular adhesion protein-1 levels and circulating cardiac

# marker levels

|  | **Correlation coefficient** | ***P* value** |
| --- | --- | --- |
| NT-proBNP (pg/mL) | 0.085 | 0.077 |
| BNP (pg/mL) | 0.116 | 0.015 |
| MMP-2 (ng/mL) | 0.440 | <0.001 |
| Galectin-3 (pg/mL) | 0.154 | 0.001 |
| hsCRP (ng/mL) | -0.099 | 0.040 |

*NT-proBNP, N-terminal pro-B-type natriuretic peptide; BNP, brain natriuretic peptide; hsCRP, high-sensitivity C-reactive protein; MMP, matrix metalloproteinase*

**Table S2 |** Echocardiographic data of the study population

|  | Tertiles of VAP-1 levels | | |  |
| --- | --- | --- | --- | --- |
|  | Tertile 1  (n = 61) | Tertile 2  (n = 75) | Tertile 3  (n = 78) | *P* value |
| LV mass index (g/m^2^) | 118.6 ± 28.6 | 121.4 ± 41.6 | 115.4 ± 33.5 | 0.541 |
| LVDs (mm) | 32.4 ± 5.3 | 35.5 ± 10.3 | 33.8 ± 6.5 | 0.020 |
| LVDd (mm) | 50.5 ± 5.9 | 52.1 ± 6.7 | 51.7 ± 6.1 | 0.173 |
| LVESV (mL) | 37.1 ± 14.1 | 41.0 ± 22.8 | 37.1 ± 20.3 | 0.341 |
| LVEDV (mL) | 96.6 ± 29.0 | 101.9 ± 37.7 | 93.3 ± 33.7 | 0.266 |
| LVEF (%) | 63.7 ± 7.4 | 61.6 ± 8.4 | 62.5 ± 8.2 | 0.123 |
| IVST (mm) | 10.3 ± 1.9 | 10.1 ± 1.7 | 10.0 ± 1.8 | 0.628 |
| PWT (mm) | 10.4 ± 1.8 | 10.2 ± 3.4 | 10.0 ± 1.7 | 0.525 |
| E wave (cm/sec) | 68.1 ± 23.1 | 78.1 ± 27.9 | 91.0 ± 30.1 | <0.001 |
| A wave (cm/sec) | 86.4 ± 22.2 | 90.6 ± 24.0 | 91.0 ± 22.9 | 0.460 |
| E/A | 0.8 ± 0.2 | 0.9 ± 0.4 | 1.2 ± 1.4 | 0.025 |
| E’ (cm/sec) | 6.1 ± 2.0 | 6.1±1.7 | 6.3 ± 1.6 | 0.633 |
| E/E’ | 12.0 ± 5.1 | 13.7 ± 5.6 | 14.9 ± 5.6 | 0.009 |
| LA dimension (mm) | 39.9 ± 6.9 | 41.5 ± 7.0 | 42.0 ± 6.8 | 0.127 |

*VAP,* vascular adhesion protein; *LV*, left ventricle; *LVDs*, left ventricular end-systolic diameter; *LVDd*, left ventricular end-diastolic diameter; *LVESV*, left ventricular end-systolic volume; *LVEDV*, left ventricular end-diastolic volume; *LVEF*, left ventricular ejection fraction; *IVST*, interventricular septal thickness in diastole; *E*, peak early diastolic flow velocity; *A*, peak late diastolic flow velocity; *PWT*, LV posterior wall thickness in diastole; *LA*, left atrium.
